# Fluorodeoxyglucose-positron emission tomography scan-positive recurrent papillary thyroid cancer and the prognosis and implications for surgical management

**DOI:** 10.1186/1477-7819-10-192

**Published:** 2012-09-17

**Authors:** Jennifer MJ Schreinemakers, Menno R Vriens, Nuria Munoz-Perez, Marlon A Guerrero, Insoo Suh, Inne HM Borel Rinkes, Jessica Gosnell, Wen T Shen, Orlo H Clark, Quan-Yang Duh

**Affiliations:** 1 Department of Surgery, University of California, 1600 Divisadero Street, Box 1711, San Francisco, CA, 94115, USA; 2 Department of Surgery, University Medical Center Utrecht, Heidelberglaan 100, Utrecht, CX, 3584, the Netherlands; 3 Department of Surgery, Hospital Universitario Virgen de las Nieves, Avenida de las Fuerzas Armadas 2, Granada, 18012, Spain; 4 Department of Surgery, University of Arizona, 1501 North Campbell Avenue, Tuscon, AZ, 85724, USA

## Abstract

**Background:**

To compare outcomes for patients with recurrent or persistent papillary thyroid cancer (PTC) who had metastatic tumors that were fluorodeoxyglucose-positron emission tomography (FDG-PET) positive or negative, and to determine whether the FDG-PET scan findings changed the outcome of medical and surgical management.

**Methods:**

From a prospective thyroid cancer database, we retrospectively identified patients with recurrent or persistent PTC and reviewed data on demographics, initial stage, location and extent of persistent or recurrent disease, clinical management, disease-free survival and outcome. We further identified subsets of patients who had an FDG-PET scan or an FDG-PET/CT scan and whole-body radioactive iodine scans and categorized them by whether they had one or more FDG-PET-avid (PET-positive) lesions or PET-negative lesions. The medical and surgical treatments and outcome of these patients were compared.

**Results:**

Between 1984 and 2008, 41 of 141 patients who had recurrent or persistent PTC underwent FDG-PET (n = 11) or FDG-PET/CT scans (n = 30); 22 patients (54%) had one or more PET-positive lesion(s), 17 (41%) had PET-negative lesions, and two had indeterminate lesions. Most PET-positive lesions were located in the neck (55%). Patients who had a PET-positive lesion had a significantly higher TNM stage (*P* = 0.01), higher age (*P* = 0.03), and higher thyroglobulin (*P* = 0.024). Only patients who had PET-positive lesions died (5/22 vs. 0/17 for PET-negative lesions; *P* = 0.04). In two of the seven patients who underwent surgical resection of their PET-positive lesions, loco-regional control was obtained without evidence of residual disease.

**Conclusion:**

Patients with recurrent or persistent PTC and FDG-PET-positive lesions have a worse prognosis. In some patients loco-regional control can be obtained without evidence of residual disease by reoperation if the lesion is localized in the neck or mediastinum.

## Background

Papillary thyroid cancer (PTC) accounts for 80 to 85% of all thyroid cancers. Although most patients with PTC have a good prognosis, recurrent disease develops in about 20 to 30%, and about 7% of patients die from progressive disease within 10 years of diagnosis [1]. After initial surgery for PTC, follow-up consists of regular ultrasound examination of the neck, measurements of serum thyroglobulin (Tg) and Tg antibodies, and whole-body iodine scans (WBS) [2]. Patients who have recurrent or persistent disease usually have increased serum Tg levels. Recurrent PTC can be identified best by ultrasound imaging of the neck, increased basal or stimulated serum Tg levels, and whole-body iodine scans. Metastatic PTC tumors can be identified by radioactive iodine scans, because well-differentiated thyroid cells often take up and concentrate iodine [3].

The loss of iodine uptake by metastatic PTC is associated with worse survival [4]. The sites where iodine uptake is negative in patients with recurrent or persistent PTC may be localized with fluorodeoxyglucose-positron emission tomography (FDG-PET) or FDG-PET-computed tomography (FDG-PET/CT). Both FDG-PET scans and FDG-PET/CT scans are especially useful for identifying the site of recurrent or persistent disease in patients who have increased serum Tg levels and negative whole-body iodine scans [5]. The sensitivity of FDG-PET scans (63 to 95%) and FDG-PET/CT scans (66 to 98%) is similar in patients with well-differentiated thyroid cancer, increased Tg levels and negative whole-body scans [6], but FDG-PET scans (0 to 25% [7]) have much lower specificity than do FDG-PET/CT scans (81% [6]). The positive predictive value of FDG-PET/CT ranges between 92 and 100% [8,9] and the negative predictive value is about 27% [9].

Metastatic PTCs that are FDG-PET-positive, but radioactive iodine negative, often have a higher malignant histological grade than the primary tumor, a pattern that is less common for FDG-PET-negative PTCs [10]. Furthermore, PTCs that are FDG-PET positive and have a higher maximum standard uptake value (SUV max) are associated with a worse prognosis [11]. Whether the more aggressive nature of these FDG-PET-positive PTCs in the neck warrants more aggressive surgical therapy is not unknown. To address this question, we compared the outcome of patients with recurrent or persistent PTC according to whether the cancer was positive or negative on FDG-PET or FDG-PET/CT scans. We also determined the outcome and surgical management of lesions that were FDG-PET or FDG-PET/CT positive.

## Methods

### Data collection

After obtaining approval from our institutional review board, we searched the database of the California Cancer Registry to identify all patients with PTC treated at the University of California San Francisco (UCSF) between 1984 and 2008. Some of these patients were referred to UCSF because of recurrent or persistent disease after having their initial treatment at other medical centers. For all patients with recurrent or persistent PTC, we recorded data on demographics, initial stage of PTC, thyroglobulin (Tg) levels at initial presentation, location and number of recurrences, disease-free survival and outcome including Tg levels to define outcome. Persistent of recurrent disease was defined by evidence of disease on imaging studies or elevated Tg levels postoperatively or during follow-up

We further identified the subset of patients who had either an FDG-PET scan or an FDG-PET/CT scan and a whole-body radioactive iodine (^131^I) scan, and categorized them by whether they had one or more FDG-PET-avid (PET-positive) lesions or PET-negative lesions. The medical and surgical treatment and outcome of these patients were then compared. A common indication for a FDG-PET scan was an elevated blood Tg level and a negative ^131^I scan. We reviewed all reports of the FDG-PET and FDG-PET/CT scans and documented the number of FDG-PET-positive lesions and the standard uptake value of these FDG-PET-avid lesions. The sites of the metastases were also recorded.

### Statistical analysis

Data are presented as mean ± standard deviation (SD) or median and interquartile range based on the distribution of data. T-tests, Mann–Whitney U tests and chi-square tests were used as appropriate to compare groups. A *P* value <0.05 was considered statistically significant. All analyses were done using SPSS 16.0 (SPSS Inc., Chicago, IL, USA).

## Results

**Figure 1:**
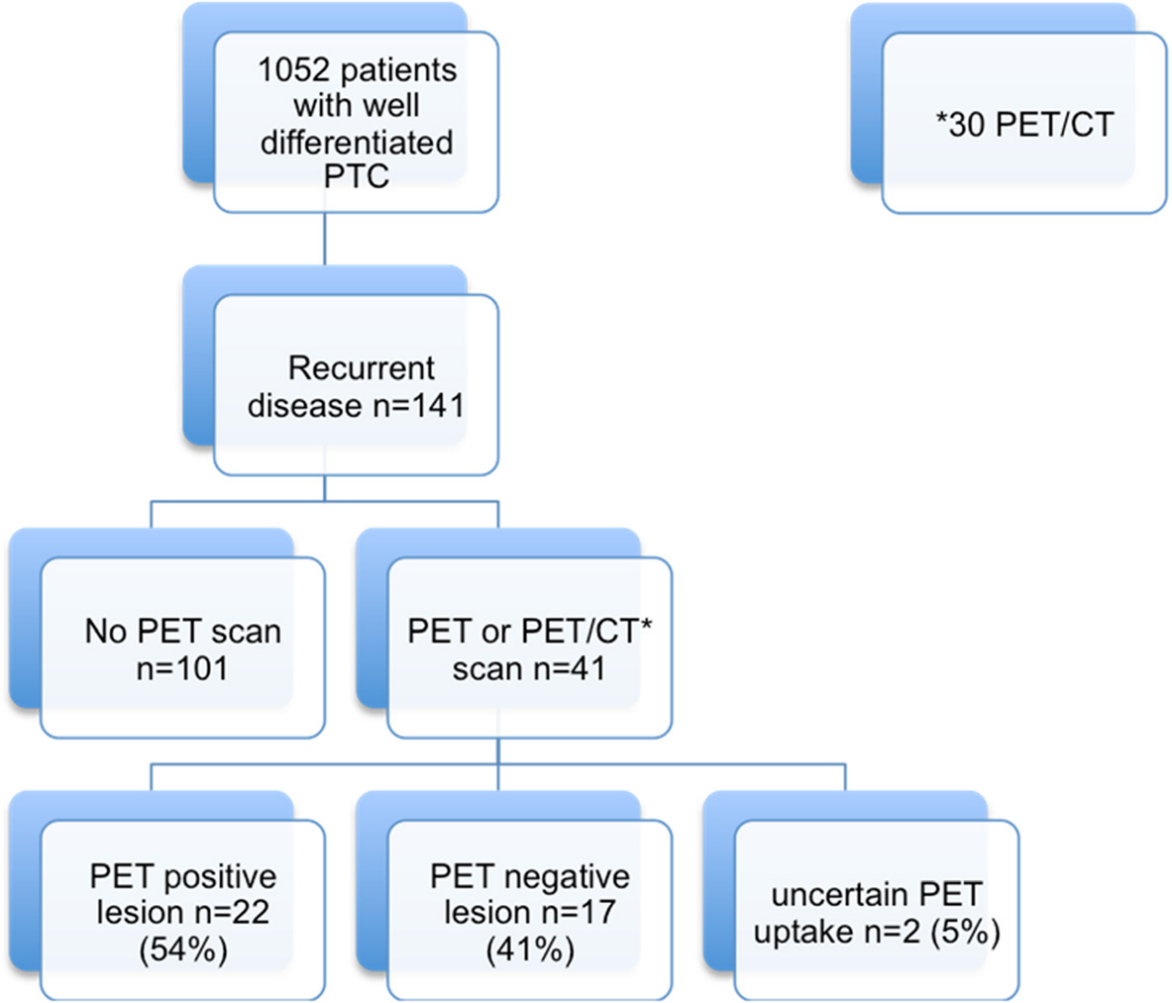
Flow chart showing patient selection and outcome for patients with PTC treated at the University of California San Francisco between 1984 and 2008.

For the 24-year period, 1052 patients with PTC were identified, 141 of whom had recurrent or persistent PTC. There were nearly twice as many women as men, and the mean age was 46 years (Table 1). Forty-one patients (29%) underwent an FDG-PET scan, of which thirty were FDG-PET/CT scans. Twenty-two patients (54%) had one or more lesions that were FDG-PET-positive, seventeen (41%) had a FDG-PET-negative lesion, and two (5%) had a lesion with uncertain uptake (Figure 1). FDG-PET-positive lesions were most often seen in the neck (41%), followed by neck and lung (25%), lungs (17%), and multiple sites (13%). The median standard uptake value was 4.20 g/ml (range 1.5 to 29).

**Table 1:** Characteristics of patients with FDG-PET-positive and FDG-PET-negative recurrent papillary thyroid cancer

**Characteristic**	**FDG-PET-positive n = 22**	**FDG-PET- negative n = 17**	***P*value**
Mean age years ± SD*	51 (±21)	44+/−21	0.03
Gender male:female	10:12	6:11	n.s.
Initial TNM stage			0.01
Stage 1	4	9	
Stage 2	0	1	
Stage 3	10	1	
Stage 4	4	3	
Unknown	4	3	
Mean tumor size (mm) ± SD	33 ± 22	24 (±13)	n.s.
Extrathyroidal extension of the tumor at diagnosis (yes/no)	8 (38%)	7 (44%)	n.s.
Lymph node metastases at diagnosis (yes/no), n (%)	15 (71%)	11 (68%)	n.s.
Median thyroglobulin level at recurrence μg/L (range)	32.9 (0.9^#^-1500)	3.1 (0.3^#^-300)	0.024

*Average age of the total 141patients with recurrent PTC is 46 ± 20 years. #Six patients had normal Tg levels at the time of recurrence. The diagnosis was made on the following imaging modalities. n = 3 recurrence on PET scan. One of them had an initial T4 tumor. n = 3 Recurrence on iodine scan n = 1, unclear, but PET-positive lesion on PET scan. FDG-PET, fluorodeoxyglucose-positron emission tomography; n.s., not significant; SD, standard deviation; TNM, tumor, node, metastasis*.*

The clinical characteristics of patients with FDG-PET-positive and FDG-PET-negative tumors are listed in Table 1. Patients with positive PET lesions were significantly older (*P* = 0.03), had higher median serum thyroglobulin levels at the time of evaluation (32.9 versus 3.1 μl/L) (*P* = 0.024) and had a higher tumor, node, metastasis (TNM) stage (*P* = 0.01). Patients with positive lesions also had larger tumors although this was not statistically different. Despite the slightly lower ^131^I uptake in patients with PET-positive lesions, patients whose lesions were ^131^I negative were treated surprisingly more frequently with ^131^I (Table 2). Disease-free survival was similar between the two groups, but only patients with PET-positive lesions died of recurrent or persistent disease (five out of twenty-two versus none out of seventeen, *P* = 0.04) (Table 2). Three of these patients died of metastatic PTC. One additional patient who died of an unknown cause and another who died of endocarditis had extensive metastatic disease. The lesions of the two patients that had uncertain uptake, both proved to be recurrent disease.

**Table 2:** Comparison of outcomes for patients with FDG-PET-positive and FDG-PET- negative recurrent papillary thyroid cancer

**Characteristic**	**FDG-PET-positive n = 22**	**FDG-PET-negative n = 17**	***P*value**
Disease-free survival, (months), median (range)	15 (5–33)	41 (4–67)	n.s.
Surgical treatment, (n)	7	7	n.s.
^131^I uptake (yes/no), n (%)	5 (23%)	8 (47%)	n.s.
^131^I uptake in PET-positive lesion, n =		7	
^131^I uptake elsewhere, n =		1	
^131^I therapy (yes/no), n (%)	10 (46%)	6 (41%)	n.s.
Outcome, n (%)			n.s.
Stable disease	5 (24%)	5 (31%)	
Loco-regional control without evidence of residual disease	2 (10%)*	2 (13%)	
Progressive disease	9 (43%)	9 (56%)	
Death^†^	5 (18%)^†^	0 (0%)^†^	
Unknown	1		

*One of the two patients had no evidence of recurrent disease, although the Tg level was 3.8 ng/l. ^†^There was a significant difference for ‘death’ between the PET + and PET– group (*P* = 0.04). ^131^I, whole-body radioactive iodine scan; FDG-PET, fluorodeoxyglucose-positron emission tomography; n.s., not significant.

Seven patients underwent surgical resection of a recurrent FDG-PET-positive lesion(s) and seven patients underwent surgical resection of a FDG-PET-negative lesion (Table 3). In one of these patients with a PET-positive lesion, loco-regional control was obtained without evidence of residual disease after a mediastinal lesion (Tg 0.2 μg/L) was resected and all scanning tests were negative seven years after reoperation. Another patient had no evidence of residual disease on an ultrasound of the neck, whole-body iodine scan and PET scan; the Tg level was 3.8 μg/L four years after PET-positive metastases were removed. One patient who had a PET-positive lesion in the neck and underwent surgical resection died of metastatic thyroid cancer. Of the patients with a FDG-PET-negative lesion who underwent surgical resection, one patient had no signs of residual disease and a Tg level of 0.9 μg/L at follow-up, indicating potential cure. Four other patients who died did not undergo surgical resection of their PET-positive lesions, primarily because of the presence of diffuse pulmonary, hepatic or bone metastasis or sites in the head and neck region that were less accessible for removal. In the PET-negative group, in two patients loco-regional control was obtained without evidence of residual disease, one after a removal of a metastatic cervical lymph node from the neck, and another with radioactive iodine therapy. The remainder of patients in both groups had stable or progressive disease (Table 3).

**Table 3:** Surgical treatment and outcome of patients with FDG-PET-avid recurrent papillary thyroid cancer

**Patient**	**Location of FDG-PET-avid lesions**	**Surgical indication**	**Operative details**	**Outcome after surgical treatment of FDG- PET-avid lesion**
1	Neck lesions, levels 2 and 3	Palpable mass, level 2/3	Dissection upper neck, left side	Persistent disease;
		FNA: metastatic PTC		Tg 56.5 μg/L,
				no more PET-positive lesions;
				ultrasound:
				multiple small nodes <1 cm
2	Two neck lesions, right side; jugular node	Palpable lymph node, right side of the neck	Right-sided neck dissection	Loco-regional control without evidence of residual disease after surgical resection of both lesions;
		Tg 33.5		low Tg levels (3.8 μg/L);
				ultrasound without evidence of suspicious lymph nodes in the neck;
				negative PET scan and a negative whole-body iodine scan after four years of follow-up
3	Neck lesion	Lesion detected on ultrasound: size 1.2 cm	Lymph node resection in the central neck	Progressive disease
		FNA: recurrent tumor		PET-positive lesions in the neck and lungs
4	Neck lesion	Palpable lesion near right clavicle.	Right neck dissection	Died
		Tg level was 63.8 μg/L.		
5	Neck and lung metastases	Lesion detected on ultrasound: size 1 cm.	Central neck dissection	Progressive disease
		Tg level was 8.6 μg/L		Tg level increased from 7.9 to 17 μg/L
				PET scan showed lung metastasis but no residual disease in the neck
6	Neck lesion; PET scan initially interpreted as negative, but later interpreted as positive	Palpable mass of 2.3 cm near the left mandible, also shown by ultrasound	Several small nodes were resected and the large node	Persistent disease
		FNA: recurrent tumor		
7	Solitary mediastinal lesion	Lesion also seen on MRI, size: 1.8 x 1.3 cm	Mediastinal lymph node dissection, followed by radioactive iodine	Loco-regional control without evidence of residual disease: Tg level decreased to 0.2 μg/L up until seven years of follow-up;
		Tg level was 12 μg/L		ultrasound showed no residual disease in neck;
		No ultrasound		CT scan two years after treatment showed no evidence of pulmonary metastasis

FDG-PET/CN, fluorodeoxyglucose-positron emission tomography/computed tomography; FNA, fine needle aspiration; MRI, magnetic resonance imaging; PTC, papillary thyroid cancer; Tg, thyroglobulin.

## Discussion

FDG-PET and FDG-PET/CT can help identify metastatic thyroid cancer in patients with recurrent or persistent PTC who have increased serum thyroglobulin levels and negative or equivocal whole-body iodine scans [3,8,12]. Metastatic PTCs that lose the ability to concentrate radioactive iodine are believed to be clinically more aggressive and have a worse outcome, probably because they are less well-differentiated and cannot be treated successfully with radioactive iodine therapy. Papillary thyroid cancers that are radioactive iodine negative take up FDG-PET more readily because of their higher metabolic activity [3].

When we compared the outcome of patients with recurrent or persistent PTC who had FDG-PET-positive or FDG-PET-negative metastatic tumors, we found that patients who had positive lesions had larger tumors at the time of initial treatment. Furthermore, in these patients the initial TNM stage was higher than in patients who had a negative lesion. These patients also had higher serum thyroglobulin levels at the time of the FDG-PET scan. In our cohort study, only patients with FDG-PET-positive lesions died. Although our sample size is relatively small, this observation supports previous reports that PET-positive lesions are associated with a worse prognosis [3]. One of those previous studies showed that patients with PET-positive lesions were also older, had a higher initial cancer stage and higher thyroglobulin levels [11]. These risk factors indicate that PET-positive patients have extensive and more aggressive PTC and are more likely to have either recurrent or persistent thyroid cancer. Another study found that most patients with PET-positive metastases also had high-risk clinical characteristics like extensive extrathyroidal extension of the primary tumor, and that 70% of patients with PTC had less well-differentiated metastases than found in their primary tumors [10].

In our study, in only two of seven patients with PET-positive neck lesions who underwent reoperation loco-regional control was obtained without evidence of residual disease. One of these patients had a mediastinal lymph node metastasis resected. The other patient had two lymph nodes resected and had no evidence of residual disease on imaging studies four years after surgery, but the thyroglobulin level remained elevated. Although one might question why palliative reoperations were done in patients with distant metastasis, we believe that surgical resection of PET-positive lesions in the neck is indicated to control loco-regional disease. One might anticipate that PET-positive lesions would grow faster and be more likely to cause local morbidity. We realize that there could be a bias in our series. It is known that patients with FDG-PET-positive lesions have more aggressive disease and, therefore, the FDG-PET scans may have been performed more readily in patients suspected of more aggressive disease. Although we failed to show a clear benefit of surgical resection in this study, we believe that resection of recurrent or persistent neck lesions should usually be performed especially for recurrent tumors that are growing or are larger than 1 cm.

In our study, all FDG-PET-positive lesions that were resected were either palpable or seen on an ultrasound of the neck or magnetic resonance image (MRI) of the mediastinum. Ultrasound is generally more sensitive for identifying nodal metastases in the neck than FDG-PET and FDG-PET/CT [13]. Ultrasound-guided needle biopsy can provide a definitive diagnosis. In two patients, the decision to operate was made regardless of the FDG-PET scan result.

In a previous study, the impact of FDG-PET/CT for recurrent PTC on the clinical management changed the treatment plan in 40% of 33 patients, supported the treatment plan made before the PET scan in 27%, and failed to contribute to the management in 33% [9]. FDG-PET/CT scanning was most useful when the thyroglobulin levels were greater than 10 ng/ml. In that study, 22 out of 30 patients were treated surgically. However, they did not show the outcome of these patients. Another study that evaluated the clinical value of FDG-PET scans in 37 patients who had an increased thyroglobulin level and a negative whole-body iodine scan found that 28 patients had a positive PET scan. In 29 patients, the management of these patients appears to have changed from the initial treatment plan (23 underwent surgery, 14 of whom were disease-free after a mean of six months [14]. FDG-PET/CT scan may contribute to the surgical strategy in patients with persistent or recurrent PTC.

In our study, 23% of patients with PET-positive lesions had metastatic lesions that took up radioactive iodine. This is a lower percentage than the 37.5% reported previously [11]. In that study, prognosis was surprisingly worse in patients who had both PET-positive lesions and radioactive iodine uptake, and survival was similar, whether their lesions did or did not take up radioactive iodine [11]. We were surprised to find that among our patients who had PET- positive lesions that were less likely to take up radioactive iodine, yet more were treated with radioactive iodine than patients with PET-negative lesions. In a report of the Memorial Sloan Kettering Cancer Center experience, patients with PET-positive lesions were treated with higher doses of radioactive iodine than those with PET-negative lesions. Despite this higher dose of radioactive iodine, subsequent FDG-PET uptake was similar after one year, the PET volume of the lesions was larger, and the thyroglobulin level was also higher after one year of follow-up [15].

The results of reoperations of patients with PET-positive lesions have been studied before by Mirallie *et al.* In that series, patients with recurrent differentiated thyroid cancer and FDG-PET-positive lesions underwent surgical resection. Half of these patients had no evidence of residual disease. A subgroup of these patients even had no detectable thyroglobulin levels indicating cure [16]. However, the prognosis of patients with FDG-PET-positive lesions and PDG-PET-negative lesions after surgery remains unclear. Nevertheless, our study had several limitations: its retrospective design, relatively small sample size, and inability to obtain histological confirmation for all FDG-PET and FDG-PET/CT lesions. Moreover, using FDG-PET scanning to localize recurrent or persistent PTC has some established disadvantages. First, it has relatively low sensitivity, specificity and positive predictive value. Second, it is expensive. Third, the decision to operate does not always depend on the scan results, which are certainly of most value in patients who have elevated thyroglobulin levels, but are radioactive iodine negative and also have other negative scans. However, FDG-PET scanning can document patients with multiple distant metastases who are not candidates for reoperation.

## Conclusion

In conclusion, patients with recurrent or persistent PTC that are FDG-PET scan positive have a worse prognosis, although some can be potentially cured by reoperation after resection of metastatic tumors in the neck or mediastinum. We believe that localized neck lesions that are FDG-PET scan positive should be resected.

## Competing interests

The author(s) declare that they have no competing interests.

## Authors’ contributions

JS participation in conception and design, collected data, carried out statistical analysis and drafted the manuscript. MV participation in conception and design, analysis of data, and revision of the manuscript. NMP collected data and drafted the manuscript. MG participation in conception and design, analysis of data and revision of manuscript. IS analysis of data and drafted the manuscript. JG participation in conception and design, review of analysis and revision of manuscript. WS participation in conception and design, review of analysis and revision of manuscript. OC participation in conception and design, review of analysis and revision of manuscript. QYD participation in conception and design, review of analysis and revision of manuscript. All authors read and approved the final manuscript.
